# A full digital workflow to prefabricate an implant-supported interim restoration: case report and a novel technique

**DOI:** 10.1186/s40729-022-00455-x

**Published:** 2022-11-02

**Authors:** Yu Fu, Cuilan Yin, Shiqi Li, Dan Li, Anchun Mo

**Affiliations:** 1 State Key Laboratory of Oral Diseases, National Clinical Research Center for Oral Diseases, Department of Implantology, West China Hospital of Stomatology, Sichuan University, 14th 3 sect of Renmin South Road, 610041 Chengdu, China; 2 Department of Implantology, West China Hospital of Stomatology, Sichuan University, 14th 3 sect of Renmin South Road, 610041 Chengdu, China

**Keywords:** Immediate restoration, Surgical guide, Digital workflow, Interim restoration

## Abstract

This article describes a case with a full digital procedure to prefabricate an implant-supported interim restoration based on the preoperative digital implant planning. A fully guided surgical template is designed and printed, and then an interim restoration is fabricated based on the planned implant position through a dental computer-aided design (CAD) software. Once the implant was placed at the predetermined position through the fully guided surgical guide, the prefabricated interim restoration could be inserted immediately after the surgery, which can guide the healing of the soft tissue and enhance the esthetic outcomes. This novel technique eliminates the conventional impression making to insert an implant-supported interim restoration immediately after the implant placement surgery, which can guide the healing of the soft tissue, minimize the chairside time and optimize the clinical workflow.

## Introduction

For an implant-supported restoration of the anterior zone, a good esthetic outcome is crucial [1–4]. Providing an immediate interim restoration after an implant surgery has been suggested as a valid and predictable treatment option in the anterior esthetic zone [5, 6]. In addition, placing a well-contoured and designed interim restoration immediately after the implant placement surgery may minimize the amount of contour change of peri-implant bone and soft tissue, thus optimizing the esthetic outcomes and improving patients’ satisfaction [2].

The conventional method of fabricating an immediate interim restoration necessitates impression making after surgery, which requires additional chairside time and increases the discomfort of the patients. To minimize chairside time, a prefabricated well-contoured interim implant-supported restoration may be an alternative choice [7–9]. An implant-supported interim restoration contains crown and emergence profile. With the advances in digital technologies for implantation and restoration, digital workflows of making immediate interim restorations have been facilitated. Digital scans captured from an intraoral scanner with high precision are reported that can be used to design the virtual restoration in CAD software [10]. In addition, as for the immediate implantation, copying the tooth data from the three-dimensional object reconstructed from cone beam computed tomography (CBCT) data can also give the crown and emergence profile information for digital design [11, 12]. To make sure the fabricated interim restoration well-placed, techniques using static implant plan or dynamic navigation virtual plan to place implant based on the prosthetically driven implant plan have been reported [10, 13]. In the clinical, some methods use the prefabricated matrix-positioning device to finalize immediate interim restoration. However, the ways require the intraoral pick-up technology to finish the fabrication of the emergence profile of the restoration and the bonding to the abutment. Some methods make immediate interim restoration using a fully guided template to make immediate interim restoration on the working cast preoperatively, which confirms that the interim restoration can be bonded to abutment preoperatively. However, these ways are still time-consuming.

The article presents a case to describe an alternative digital way to fabricate an implant-supported interim restoration preoperatively followed by immediate restoration conveniently, rapidly and accurately, aiming at guiding the healing of the soft tissue, reducing chairside time and optimizing clinical workflow.

## Case report

**Fig. 1:**
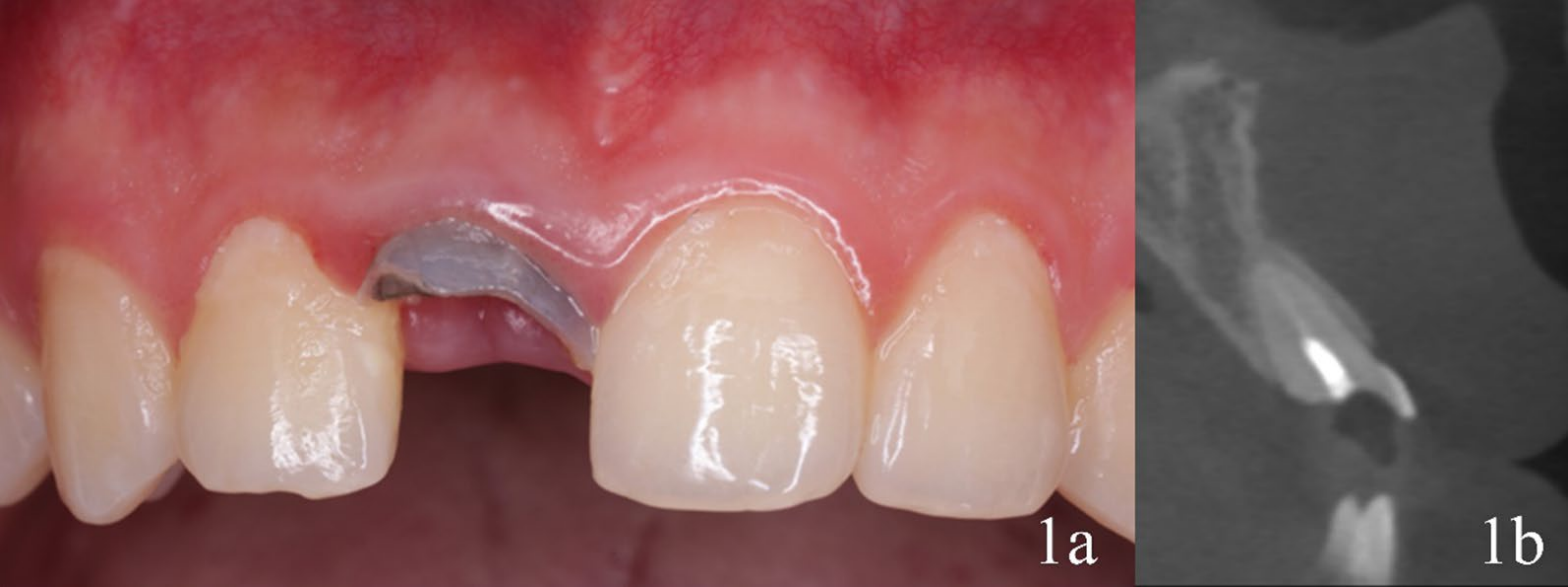
Preoperative situation. **a** Frontal view of the upper right central incisor. **b** CBCT scan of the upper right central incisor area, cone beam computed tomography

A 27-year-old male patient presented to the West China Hospital of Stomatology at Sichuan University with the fractured maxillary right central incisor due to dental trauma some years previously, requesting oral rehabilitation. The patient received no fixed restoration or removable prosthesis before seeking treatment. Clinical evaluation revealed fractured maxillary right central incisor with tooth discoloration, where the lowest point of the crown is lying sub gingival, while the radiographic examination revealed that the tooth was a root canal treated tooth without any periapical involvement and the labial bone wall was complete (Fig. 1). The patient came from another place who was keen for the fewest dental visits and expected earliest possible restoration of his teeth. Systemic examination of the patient revealed no hypertension, cardiopathy, or diabetes and routine blood investigation was carried out to exclude any other related disorders. Considering the unfavorable prognosis, the treatment alternatives were discussed with the patient and extraction followed by immediate implant placement and an interim restoration with a digital workflow was accepted.

**Fig. 2:**
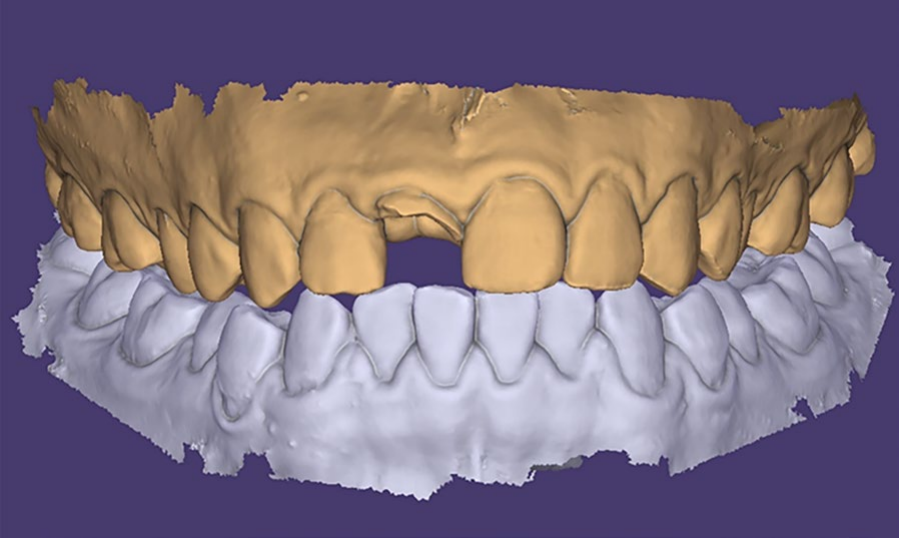
Scan data of maxillary and mandibular-arches

**Fig. 3:**
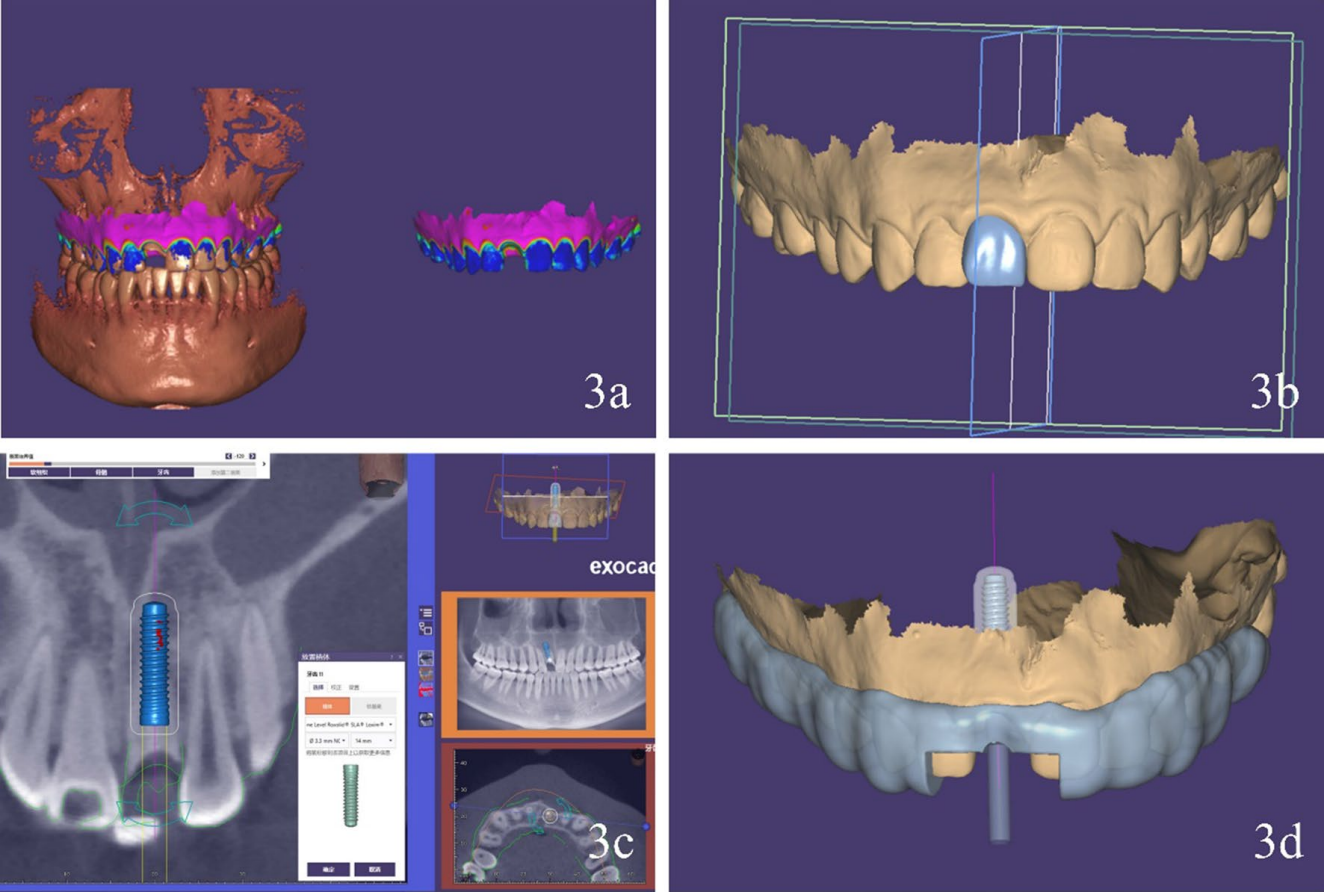
Digital surgical guide design. **a** Intraoral scan and CBCT scan superimposed. **b** Design of diagnostic virtual interim restoration. **c** Placement of virtual implant. **d** Completed surgical guide. *CBCT* cone beam computed tomography

**Fig. 4:**
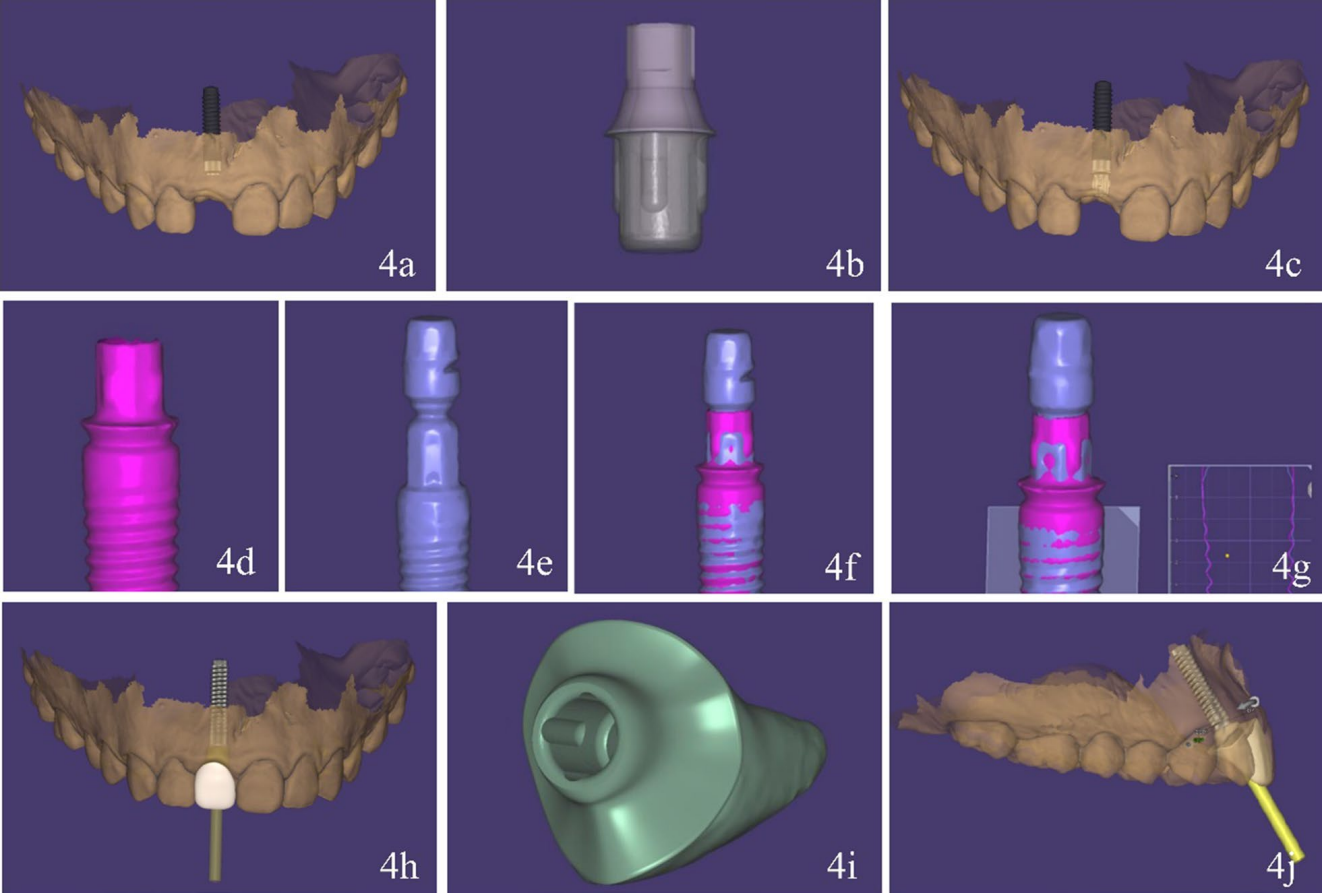
Design of implant-supported interim restoration based on designed implant position. **a** Scene files loaded. **b** selected virtual abutment (Straumann NC, Variobase, Straumann USA). **c** Virtual abutment selected. **d** Scan of the implant screwed with abutment. **e** Scan of the implant screwed with implant carrier. **f** Two scans were superimposed to find out the direction relationship between the implant carrier and the abutment to help bonding the crown to the abutment. **g** Accuracy of the superimposing process was reliable. **h** Design of implant-supported interim restoration based on selected virtual abutment. **i** Concave emergence profile designed. **j** interim restoration fit to the abutment

**Fig. 5:**
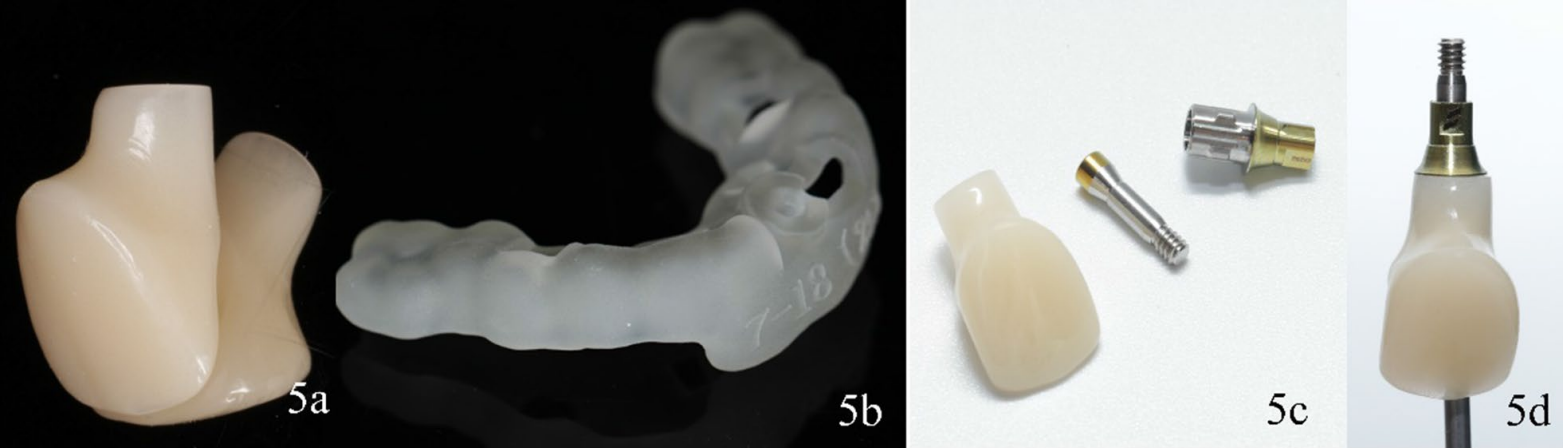
Fabrication of interim restoration and surgical guide. **a** Interim restoration milled. **b** Surgical guide printed; cementation of interim restoration and abutment. **c** Interim restoration and abutment. **d** Bonding the center of the buccal side of the crown with facing the concave plane of the cross-fit structure of the abutment

This clinical report describes a digital workflow to prefabricate an implant-supported interim restoration, followed by implant placement and immediate restoration conveniently, rapidly and accurately. Therefore, the technique was devised and the specific procedure is as follows:

1. Use an intraoral scanner (CS-3600, Carestream) to obtain optical maxillary and mandibular intraoral scans and the interocclusal registration (Fig. 2). Export the digital data in standard tessellation language (STL) file format.
2. Establish a treatment plan for implant placement and surgical guide design using a dental computer aided design (CAD) software (exocad Dental Implant; exocad GmbH). Import the patient’s DICOM files and load the intraoral scan data into the same software. First superimpose the maxillary arch to the CBCT data using three reference points and “best fit” (Fig. 3a). Design a virtual interim restoration without emergence profile at the implant site (Fig. 3b). Then, place a virtual implant (Straumann NC, Bone Level, guided 3.3 mm × 14 mm; Straumann USA) (Fig. 3c) driven by the diagnostic interim restoration and then design a fully guided surgical template (Fig. 3d) based on the three-dimensional position of the virtual implant. Save the scene files, rename it and change its format from “implant planning scene” to “dental CAD”.
3. Establish a new order for designing the implant-supported interim restoration at the site of virtual implant placed in a CAD software (exocad Dental Implant; exocad GmbH). Transmit the modified scene files (containing the information of virtual diagnostic casts and the planned implant locations) to the new order file. Load the scene files in the same CAD software (Fig. 4a). Select one abutment (Straumann NC, Variobase, Straumann USA) to apply to the implant (Fig. 4b, c) which is the key part of this technique. First, scan the implant screwed with abutment. On top of that, scan the implant screwed with implant carrier the data of which can be found in the CAD software. Then, superimposed the two scans to find out the direction relationship between the implant carrier and the abutment (Fig. 4d–g). We should use the laser marking point of the implant carrier to rotate the implant in the right direction, helping to bond the crown to the abutment through the laser marking point on the buccal side.
4. Design the implant-supported interim restoration and remove all occlusal contacts in centric or eccentric occlusion and proximal contacts, based on the virtual abutment’s position and the intraoral scans (Fig. 4h). Shape the emergence profile according to the following guidelines. As we know, emergence profile can be divided in two areas, which are the critical contour and subcritical contour [14]. The critical contour is maintained palatally and interproximally just like natural tooth, while the facial portion should be trimmed down to 0.5–1 mm to match the postoperative gingival recession. The subcritical contour is shaped as concave as possible to avoid any compression on soft and hard tissue (Fig. 4i). [15] And then add a hole fit to the screw channel of the abutment (Fig. 4j).
5. Fabricate the implant-supported interim restoration and surgical guide (Projet MJP 3600; 3D system) (Fig. 5a, b). Bond the milled interim restoration to the anti-rotational abutment (Straumann NC, Variobase, Straumann USA) with the center of the buccal side of the crown is facing the concave plane of the cross-fit structure of the abutment (Straumann NC, Variobase, Straumann USA) before surgery using composite resin (Filtek Supreme Ultra; 3MESPE) (Fig. 5c, d).

**Fig. 6:**
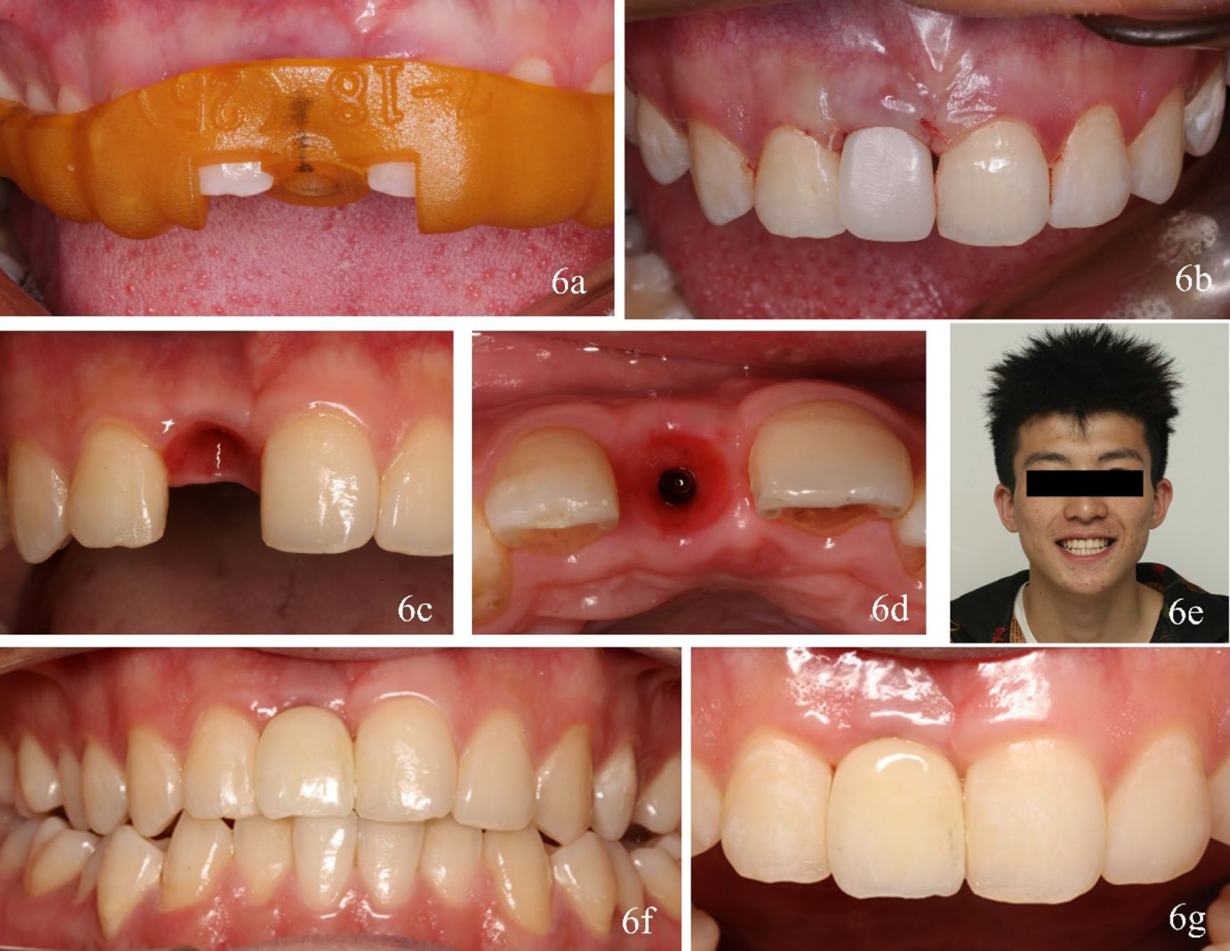
Implant surgery, (**a**) surgical guide seated. **b** Placement of immediate interim restoration after implant surgery. Soft-tissue emergence profiles 3 months after placement, **c** frontal view, **d** occlusal view. Definitive crown seated, **e** facial view of the patient. **f**–**g** Frontal view of the final prosthesis

In the operation, maxillary right central incisor was extracted first and the implant placement surgery was carried out. With the guidance of the fully guided surgical template, the implant (Straumann NC, Bone Level, guided 3.3 mm × 14 mm; Institute Straumann AG, Straumann USA) was placed in the planned position by making sure the laser marking points of the implant carrier facing the buccal side (Fig. 6a). Adequate primary stability was obtained. Insert the prefabricated interim restoration and tighten it to 15 N·cm. Use PTFE tape and composite resin (Filtek Supreme Ultra; 3MESPE) to seal the screw access hole and remove all occlusal contacts in centric and eccentric occlusal movement (Fig. 6b). The postoperative CBCT was recorded to show the actual implant position. The implant was well-positioned to support this temporary fixed prosthesis.

Three months after interim restoration placed, the patient returned for the future definitive crown. The intraoral outcomes of the maturation of the peri-implant soft tissues showed good healing without any complications and peri-implant health was maintained (Fig. 6c, d). Screw-retained zirconia single crown was fabricated and restored the missing tooth (Fig. 6e–g). The occlusal force of the prosthesis was adjusted to be less than that of the natural tooth. The functional and esthetic result was accepted by the patient.

**Fig. 7:**
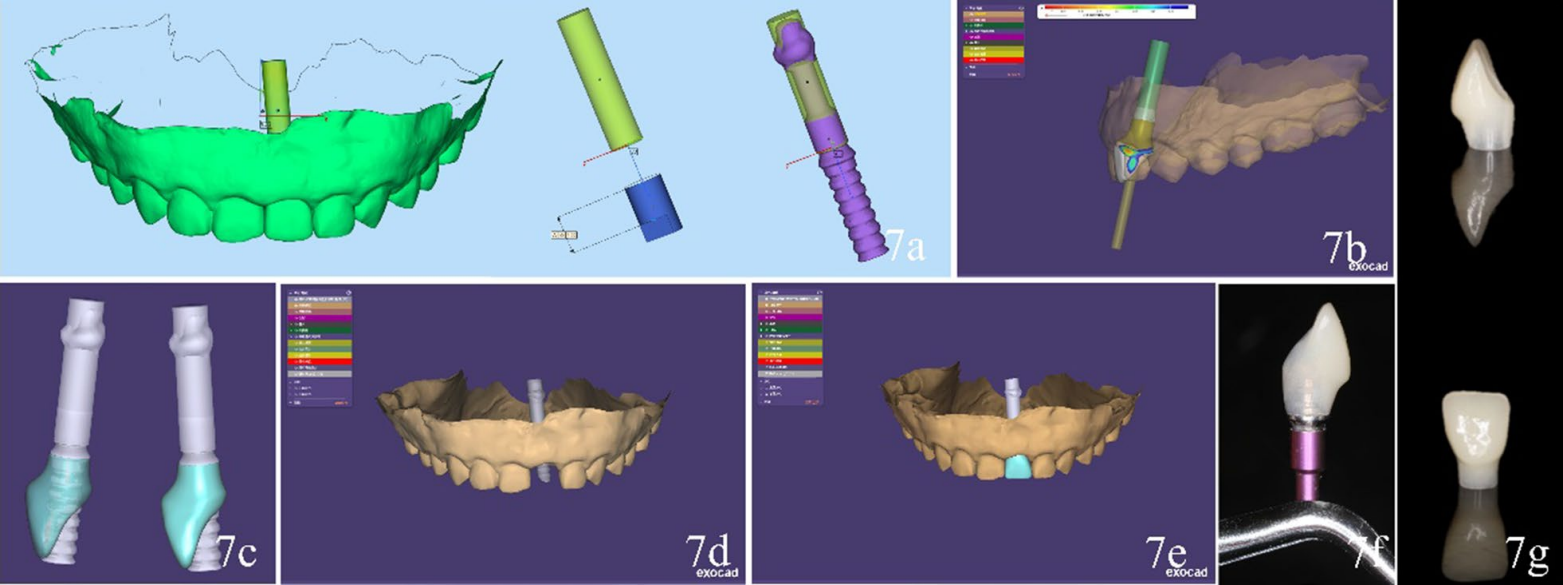
Fabrication of implant-supported interim restoration based on designed implant position. (**a**) Alignment of digital data of interim abutment with the vital implant and implant scanning arm. **b**–**e** Design of implant-supported interim restoration based on selected virtual abutment. **f** Bonding the center of the buccal side of the crown with facing one side of the regular hexagon of the abutment. **g** Interim restoration milled

**Fig. 8:**
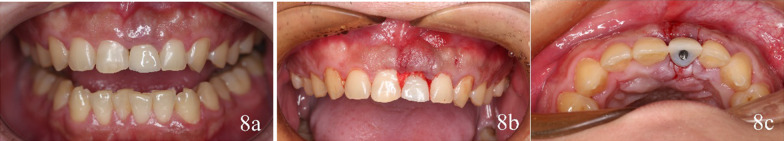
**a** Pre-operation intraoral view. Placement of immediate interim restoration after implant surgery, **b** frontal view, **c** occlusal view

It should be pointed out that the above technique can be applied to any dental implant system if the CAD software possesses the corresponding temporary abutment data. Take another case as an example. Briefly, after superimposing the preoperative CBCT scan data and intraoral scan data in Clinician software, one implant (NobelActive NP, 3.5 × 15 mm; Nobel Biocare) was placed virtually. The scene files were saved. Then, a fully guided surgical was designed and printed three-dimensionally. Data of the virtual implant and diagnostic casts with the restoration were imported into the CAD software as STL format. Due to the encryption, the implant appeared as a cylinder in yellow of the same size. According to the position relation between the implant and implant scanning arm, an interim abutment (Temporary Snap Abutment NP, Engaging; Nobel Biocare) was attached to the virtual implant on the platform and axis (Fig. 7a). Then, interim restoration was designed on the interim abutment and the emergence profile was shaped to a concave curve as per the soft-tissue margin and implant depth (Fig. 7b, c), in other words, the most superficial area supported the existing gingival margin and the deeper area allowed a space for reconstructing soft tissue [15]. After that, the excess length of the interim abutment was trimmed away virtually (Fig. 7d, e). Finally, the interim restoration was fabricated and bonded to the interim abutment trimmed as designed (Fig. 7f, g). After the implant placement surgery, the implant-supported interim restoration was inserted perfectly (Fig. 8a–c).

## Discussion

The applications of digital technologies in implantation and restoration enable the fabrication of an interim restoration preoperatively. In this article, a full digital technique to prefabricate an implant-supported interim restoration was devised. First, preoperative CBCT scan and intraoral scan should be recorded to generate a virtual cast offering bone and soft tissue conditions of the patient. Second, a diagnostic trial restoration is designed. Then, the implant planning is made through superimposing cone beam computed tomography (CBCT) data with diagnostic trial restoration data. Subsequently, the surgical guide and interim restoration can be designed and fabricated by the surgical guide planning. After the implant being inserted into the predetermined position assisted by fully guided surgical template, the interim restoration which was bonded to the interim abutment prior to the implant placement can be seated well. In the above cases, the fully guided surgical guide assisted fabrication of the interim prosthesis and insertion of the implant, thus the placement of the interim restoration can be successful, which in turn proves the accurate placement of the implant.

The conventional method of fabricating an immediate interim restoration needs impression making after surgery, and fabricating the restoration which requires additional chairside time or CAD/CAM machine and increases the discomfort of the patients. Another way to make an immediate interim restoration is reported using the natural tooth extracted at the implant site. However, it cannot be bonded to the abutment directly, which is still time consuming. Making interim restorations copying the contour of the original natural teeth to contouring the peri-implant soft tissue is commonly accepted by technicians [11, 12]. However, this technique requires the tooth to be extracted to have an ideal residual anatomic structure, which cannot always be achieved. Moreover, because the transgingival zone of an implant is significantly different from a natural tooth and the three-dimensional position of an implant influence the design of the emergence profile part of the protheses, simply mirror or copy the tooth to be extracted does not always get a stable and esthetic result. In addition, it is reported that the prefabricated matrix-positioning device can be used to finalize immediate interim restoration. However, it requires bonding the restoration and abutment intraorally and it requires additional chairside time. In addition, in the pick-up process, the residual and release of the monomer of the resin may increase the risk of infection in the surgery area, which also affects the reformation of soft tissue [9].

To avoid these shortcomings, another report used the fully guided surgical template and abutment guide lock to fabricate the immediate interim restoration extraorally. This technique still costs a lot of time before surgery and needs to fabricate the emergence profiles using self-solidification resin followed by shaping and polishing the emergence profile manually. However, the prefabricated interim restoration in this article was totally CAD/CAM fabricated, significantly reducing the chairside time compared that in the upper ways [13]. In addition, the emergence profiles were also CAD/CAM fabricated, which were shaped precisely with smooth surface to avoid the polishing procedure. The critical area was modified equal to the natural tooth. The subcritical area was shaped as concave as possible to obtain optimal results in the peri-implant soft tissues, which is quite important to allow a space for regenerative process.

What’s more, adjustment of occlusal and proximal contacts was suggested to be taken into consideration, because most scholars believe that these contacts will cause micromotion on bone–dental implant interface during postsurgical healing, which disturbs the implant osseointegration process [16, 17]. Therefore, it is not advisable to fully replicate the anatomical and esthetic characteristics of the homonym teeth. In addition, shaping the emergence profile slightly concave is essential for soft tissue to get adequate space to grow and, moreover, to obtain soft tissue sealing [18–21].

In this report, a well-shaped interim prosthesis with a concave subgingival contour based on the precise position of implant is fabricated to enhance the tissue thickness and optimize the peri-implant soft tissue architecture. It should be mentioned that the crown should be boned to the correct direction of the anti-rotational abutment to make sure the placement of the interim restoration. Making sure the direction relationship among the implant, implant carrier, abutment, and crown is curial for the bonding process.

The technique has limitations. If the accuracy of surgical guide fabricated from the virtual treatment plan is poor, the implant cannot be inserted into the preplanned position, then the interim crown will require more adjustments. In addition, the digital designed individual crown and emergence profile may not be ideal, requiring some manual adjustments. On the other hand, this technique will be of no use if the facility does not have relative software and sufficient database.

## Conclusions

This technique article offers a full digital procedure to preoperatively design and fabricate an interim implant-supported restoration in the esthetic zone. Fully guided surgical guide is used to assist virtual design of the well-contoured interim prosthesis, and accurate implant placement providing the conditions for precise insertion of the interim restoration.

## Data Availability

Not applicable.
